# Supplementary material for: Comparative genomics of Mycobacterium africanum Lineage 5 and Lineage 6 from Ghana suggests distinct ecological niches
Source: Sci Rep. 2018 Jul 26;8:11269. doi: 10.1038/s41598-018-29620-2 (PMC6062541; doi:10.1038/s41598-018-29620-2)
Supplement: Supplementary file 1 — Supplementary Information [file 41598_2018_29620_MOESM1_ESM.pdf]

Comparative genomics of *Mycobacterium africanum* Lineage 5 and Lineage 6 from Ghana suggests different ecological niches.

Authors:

Isaac Darko Otchere[1,2γ], Mireia Coscollá[3,4γ], Leonor Sánchez-Busó[5], Adwoa Asante-Poku[1], Daniela Brites[3,4,] Chloe Loiseau[3,4,] Conor Meehan[6], Stephen Osei-Wusu[1], Audrey Forson[7], Clement Laryea[8], Abdallah Iddrisu Yahayah[9], Akosua Baddoo[7], Gloria Akosua Ansa[10] Samuel Yaw Aboagye[1], Prince Asare[1], Sonia Borrell[3,4], Florian Gehre[6,11], Patrick Beckert[12,13], Thomas A Kohl[12,13], Sanoussi N'dira[14], Christian Beisel[15], Martin Antonio[11], Stefan Niemann[12,13], Bouke C de Jong[6,11], Julian Parkhill[5], Simon R Harris[5], Sebastien Gagneux[3,4]*, Dorothy Yeboah-Manu[1]*.

Author Affiliations:

[1]Noguchi Memorial Institute for Medical Research, University of Ghana, Legon, Accra, Ghana.

[2]Department of Biochemistry, Cell and Molecular Biology, University of Ghana, Legon, Accra, Ghana.

[3]Swiss Tropical and Public Health Institute, Basel, Switzerland.

[4]University of Basel, Basel, Switzerland.

[5]Wellcome Trust Sanger Institute, University of Cambridge, Hinxton, United Kingdom.

[6]Institute of Tropical Medicine, Antwerp, Belgium

[7]Chest Clinic, Korle-Bu Teaching Hospital, Accra, Ghana.

[8]37 Military Hospital, Accra, Ghana.

[9]Chest Department, Tamale Teaching Hospital, Tamale, Ghana.

[10]Public Health Department, University of Ghana Hospital, Legon, Accra, Ghana

[11]Medical Research Council, The Gambia Unit, Gambia

[12]Molecular and Experimental Mycobacteriology, Research Center Borstel, Borstel, Germany

[13]Germany German Center for Infection Research, Partner Site Hamburg-Borstel-Lübeck, Germany

[14]National Reference Laboratory for Mycobacteria, Cotonou, Benin

[15]Department of Biosystems Science and Engineering, ETH Zurich, Basel, Switzerland.

*These authors contributed equally

[γ]These authors contributed equally

Correspondence should be addressed to: DYM (dyboah-Manu@noguchi.ug.edu.gh and SG (sebastien.gagneux@swisstph.ch)

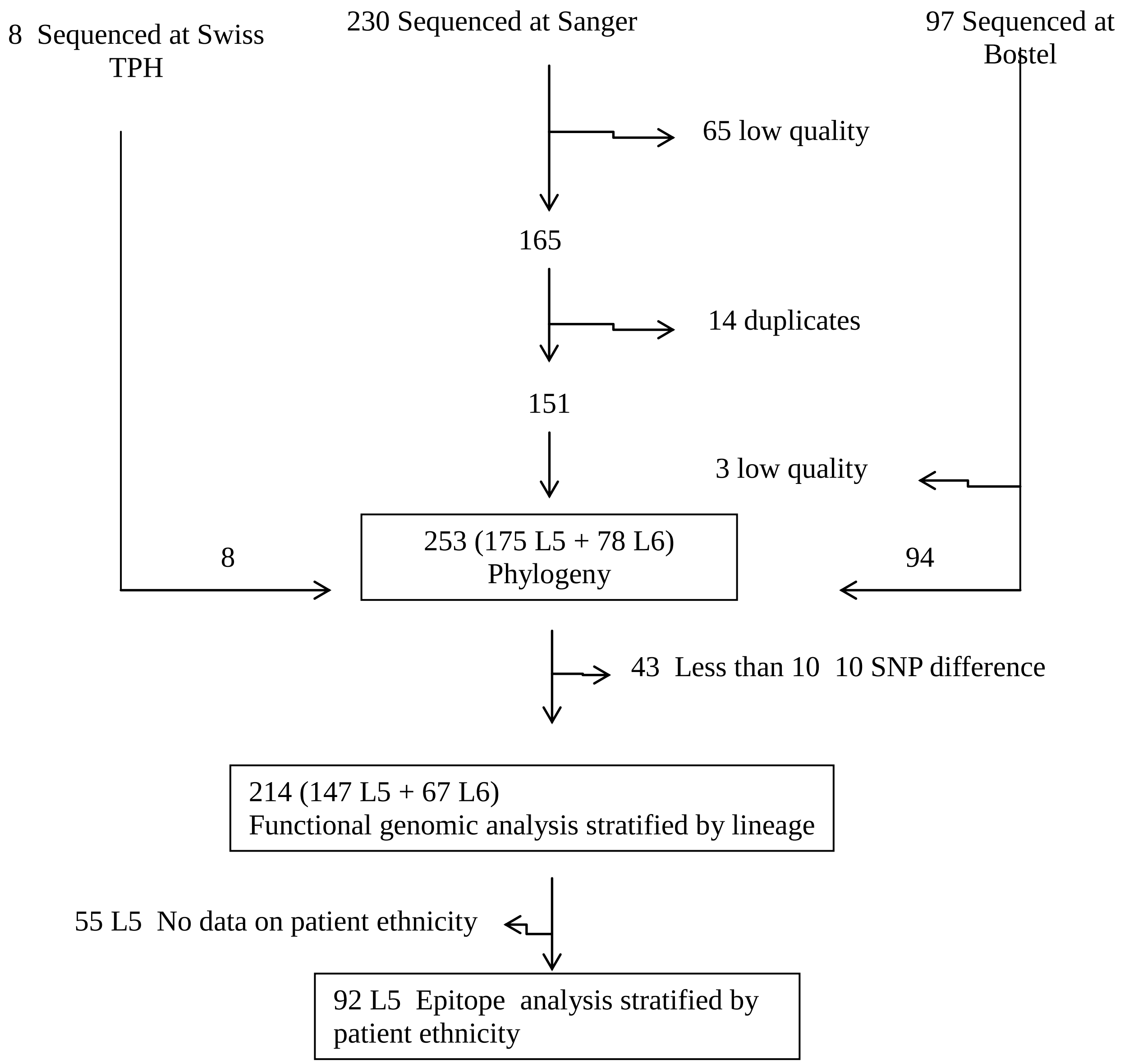

Supplementary figure S1: Flow chart of Ghanaian *Maf* genomes used for the study. Sites of DNA sequencing as well as number of genomes used for each specific analysis are indicated.

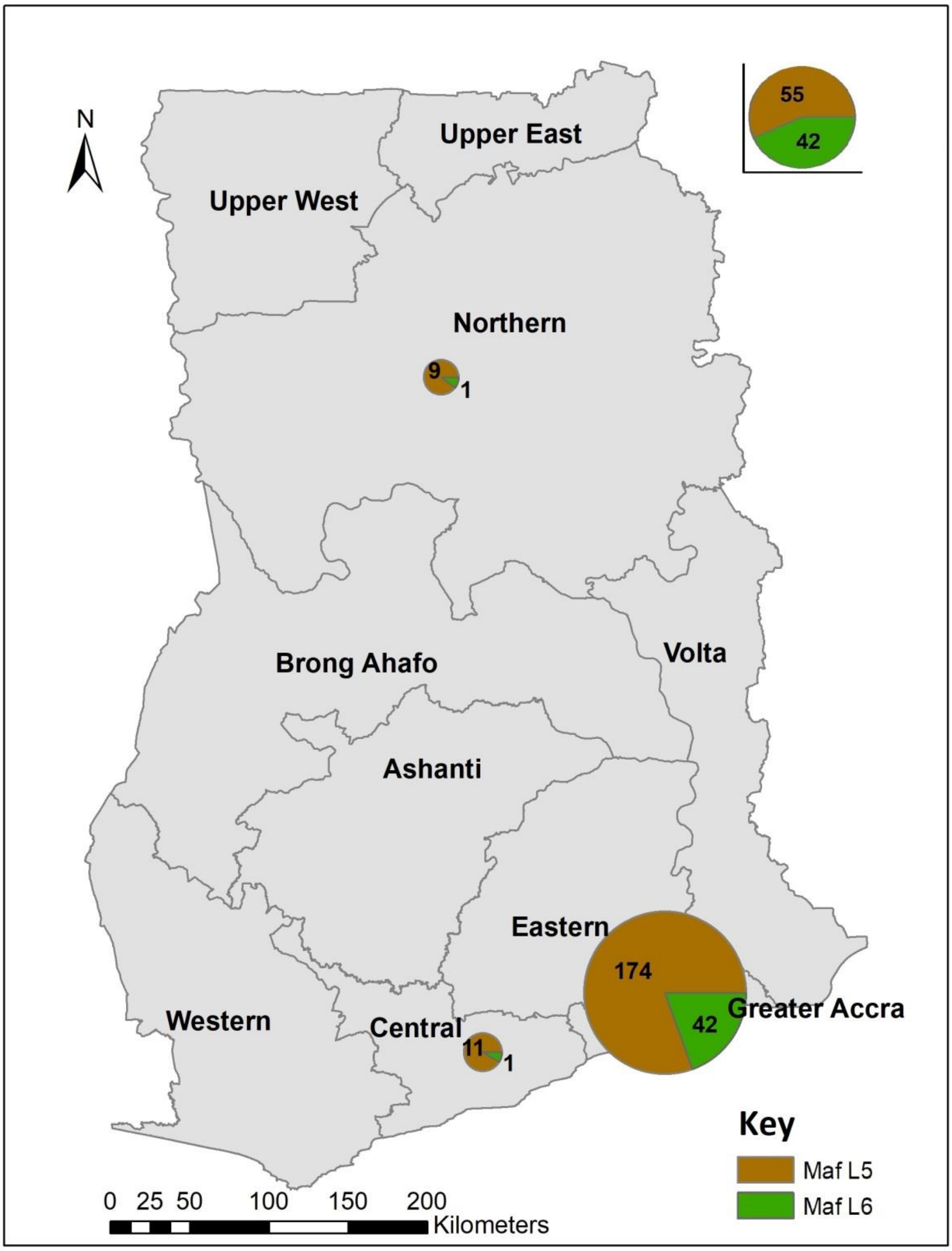

Supplementary figure S2: Map of Ghana showing the regional distribution of *Maf* isolates. The sizes of pie charts correspond to number of isolates from the respective regions. the map was generated with the ArcGIS ArcMap tool ESRI version 10.2.2 (https://support.esri.com/en/Products/Desktop/arcgis-desktop/arcmap/10-2-2).

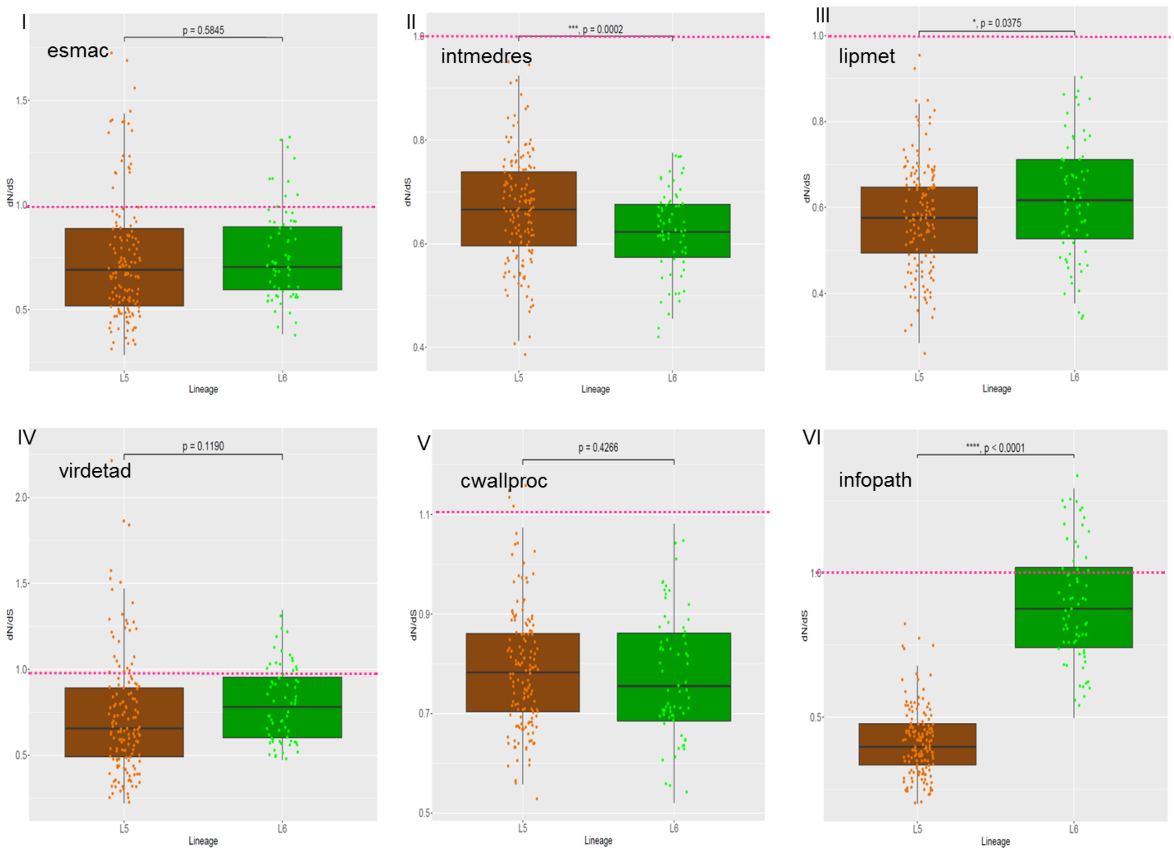

Supplementary figure S3: Pairwise dN/dS of genes of six functional categories using all the 147 L5 against the 67 L6 genomes. *esmac* – genes essential for growth in macrophages, *intmedres* – genes involved with intermediate metabolism and respiration, *lipmet* – genes involved with lipid metabolism, *virdetad* – genes involved with virulence, detoxification and adaptation, *cwallproc* - genes involved with cell wall and cell processes and *infopath* – genes involved with information pathways

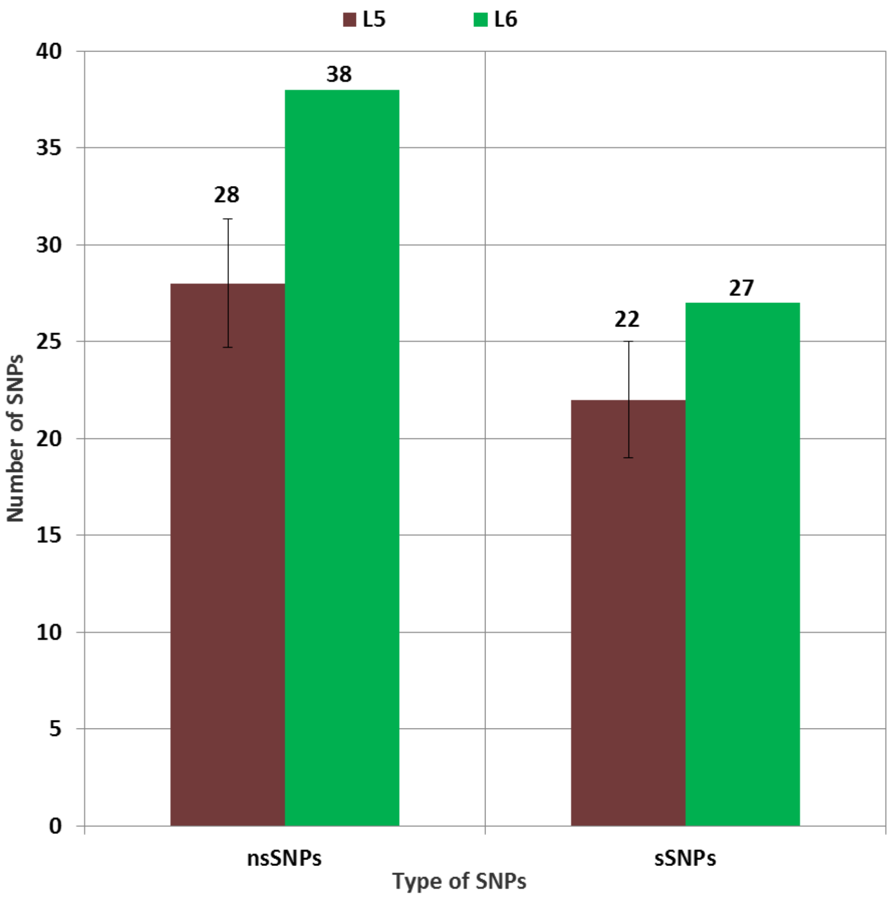

Supplementary figure S4: Number of non-redundant SNPs within human T cell epitopes stratified by *Maf* lineage. No difference (X-squared = 0.0055391, df = 1, p-value = 0.9407) between the proportion of nsSNPs between L6 (67 genomes) and L5 (mean values of 10 random samples of size = 67 with replacement)

Supplementary table S5: Mutated Human T cell antigens and epitopes of *Maf* with mutations stratified by lineage

Supplementary Table S5: Human T-cell antigens and epitopes with amino acid substitutions

Lineage	Antigen	Start	Stop	IEDB ID	Mutation	Gene product	Function
L6	Rv0206c	246311	246337	144970	L336V	Possible conserved transmembrane transport protein MmpL3	cell wall and cell processes
L5	Rv0222	266182	266235	65198	S241C	enoyl-CoA hydratase EchA1	lipid metabolism
L5	Rv0288	352058	352111	3196	M81V	Low molecular weight protein antigen 7 EsxH	cell wall and cell processes
L6	Rv0288	351860	351886	140717	P9L		
L6	Rv0288	351938	351991	12402	V34M		
L5	Rv0289	352389	352433	178694	P91Q	ESX-3 secretion-associated protein EspG3	cell wall and cell processes
L6	Rv0290	353839	353883	178433	S266R	ESX conserved component EccD3	cell wall and cell processes
L6	Rv0290	354148	354192	178957	R370C		
L6	Rv0290	354448	354492	179031	I456T		
L5	Rv0291	355809	355853	178530	V443A	Probable membrane-anchored mycosin MycP3	intermediary metabolism and respiration
L6	Rv0292	356075	356119	178499	A75V	ESX conserved component EccE3	cell wall and cell processes
L5	Rv0294	358171	358215	179284	P4S	Probable trans-aconitate methyltransferase Tam	intermediary metabolism and respiration
L6	Rv0299	363515	363559	178736	H15Y	Hypothetical protein	conserved hypotheticals
L5	Rv0522	613698	613742	178563	V231D	GAMA-aminobutyrate permease	cell wall and cell processes
L5	Rv0529	620575	620619	178614	P238S	Possible cytochrome C-type biogenesis protein CcsA	intermediary metabolism and respiration
L6	Rv0853c	950450	950494	179057	Q219R	Probable pyruvate or indole-3-pyruvate decarboxylase	intermediary metabolism and respiration

						Pdc	
L5	Rv0934	1043162	1043218	9923	P356L	Periplasmic phosphate-binding lipoprotein PstS1	cell wall and cell processes
L6	Rv0934	1042550	1042609	44020	A152D		
L6	Rv0934	1043027	1043086	3505	P310L		
L6	Rv0993	1109464	1109508	178909	V70A	UTP--glucose-1-phosphate uridylyltransferase GalU	intermediary metabolism and respiration
L5	Rv1028c	1150364	1150408	178770	F432L	Probable sensor protein KdpD	regulatory proteins
L6	Rv1045	1167928	1167972	179449	H93Y	Hypothetical protein	conserved hypotheticals
L5	Rv1174c	1305732	1305758	226572	A84T	Low molecular weight T-cell antigen TB8.4	cell wall and cell processes
L5	Rv1242	1384673	1384723	16879	S53R	Possible toxin VapC33	virulence, detoxification, adaptation
L5	Rv1255c	1402877	1402936	55530	R152H	Probable transcriptional regulatory protein	regulatory proteins
L5	Rv1503c	1694383	1694427	178753	E54Q	Conserved hypothetical protein	conserved hypotheticals
L6	Rv1617	1816669	1816695	144897	G161E	Probable pyruvate kinase PykA	intermediary metabolism and respiration
L5	Rv1866	2113587	2113658	120392	C168S	Conserved protein	lipid metabolism
L5	Rv1871c	2121642	2121686	178713	R81L	Conserved protein	conserved hypotheticals
L5	Rv1885c	2134792	2134836	178607	G27S	Chorismate mutase	intermediary metabolism and respiration
L5	Rv1886c	2134893	2134952	76455	Y306C	Secreted antigen 85-B FbpB (85B)	lipid metabolism
L5	Rv1886c	2135748	2135867	181733	G18R		
L6	Rv1908c	2155107	2155151	125317	W328L	Catalase-peroxidase-peroxynitritase T KatG	virulence, detoxification, adaptation
L6	Rv1985c	2229108	2229182	179874	R256P	Probable transcriptional regulatory protein	regulatory proteins
L6	Rv1985c	2229333	2229407	179851	A188V		
L5	Rv1987	2231035	2231103	38245	T13P	Possible chitinase	cell wall and cell processes
L5	Rv2029c	2275495	2275554	154044	L297R	6-phosphofructokin	intermediary metabolism and

						ase PfkB	respiration
L5	Rv2029c	2276215	2276274	154733	H59Y		
L6	Rv2029c	2276185	2276244	155082	S77I		
L6	Rv2094c	2353076	2353120	179580	P61L	Sec-independent protein translocase membrane-bound protein TatA	cell wall and cell processes
L5	Rv2576c	2900406	2900450	178470	H91P	Possible conserved membrane protein	cell wall and cell processes
L5	Rv2627c	2954170	2954196	144882	R188G	Conserved protein	conserved hypotheticals
L6	Rv2627c	2954314	2954358	179452	T144M		
L5	Rv2628	2955058	2955102	106659	R5Q & P6L	Hypothetical protein	conserved hypotheticals
L5	Rv2628	2955073	2955117	106713	P6L		
L5	Rv2628	2955133	2955177	106537	A35V		
L5	Rv2628	2955358	2955402	106364	P103S		
L5	Rv2819c	3126972	3127016	179215	E122A	Hypothetical protein	conserved hypotheticals
L6	Rv2855	3166042	3166086	179616	D290Y	NADPH-dependent mycothiol reductase Mtr	intermediary metabolism and respiration
L6	Rv2865	3177684	3177728	178778	P58T	Antitoxin RelF	virulence, detoxification, adaptation
L6	Rv2873	3184490	3184564	153271	V197A	Cell surface lipoprotein Mpt83	cell wall and cell processes
L6	Rv2874	3185195	3185239	179117	I130T	Possible integral membrane C-type cytochrome biogenesis protein DipZ	intermediary metabolism and respiration
L6	Rv2874	3186116	3186160	179424	S436P		
L5	Rv2875	3187075	3187149	20744	D32G	Major secreted immunogenic protein Mpt70	cell wall and cell processes
L5	Rv2958c	3311113	3311139	189566	L292S	Possible glycosyl transferase	intermediary metabolism and respiration
L6	Rv2994	3351437	3351508	120878	M65I & G76S	Probable conserved integral membrane protein	cell wall and cell processes
L5	Rv3003c	3362435	3362479	178644	A183G	Acetolactate synthase (large subunit)	Involved in valine and isoleucine biosynthesis

L6	Rv3003c	3362435	3362479	178644	A177V		
L5	Rv3010c	3368919	3368963	178438	Y304H	Probable 6-phosphofructokinase PfkA	intermediary metabolism and respiration
L6	Rv3020c	3379141	3379185	178429	A56V	ESAT-6 like protein EsxS	cell wall and cell processes
L5	Rv3024c	3383625	3383669	178477	D80A	Probable tRNA (5-methylaminomethyl-2-thiouridylate)-methyltransferase TrmU	information pathways
L6	Rv3024c	3382941	3382985	178496	E305G		
L5	Rv3296	3676775	3676870	142233	A32G	Probable ATP-dependent helicase Lhr	information pathways
L5	Rv3333c	3720465	3720509	70013	T103A	Hypothetical proline rich protein	conserved hypotheticals
L5	Rv3617	4057808	4057852	178550	L31R	Probable epoxide hydrolase EphA	virulence, detoxification, adaptation
L5	Rv3634c	4073108	4073152	178744	T159I	UDP-glucose 4-epimerase GalE1	intermediary metabolism and respiration
L5	Rv3763	4209047	4209106	105967	S20P	19 kDa lipoprotein antigen precursor LpqH	cell wall and cell processes
L6	Rv3763	4209107	4209166	18914	T40A		
L5	Rv3775	4220216	4220260	179234	W192S	Probable lipase LipE	intermediary metabolism and respiration
L5	Rv3804c	4265810	4265869	21482	N266D	Secreted antigen 85-a FbpA	lipid metabolism
L6	Rv3804c	4266161	4266229	195727	T145A		
L5	Rv3823c	4288659	4288703	178473	N946H	Conserved integral membrane transport protein MmpL8	cell wall and cell processes
L5	Rv3823c	4291281	4291307	144909	P81S		
L6	Rv3823c	4290132	4290176	179015	P454A		
L5	Rv3825c	4298772	4298816	178609	G278D	Polyketide synthase Pks2	lipid metabolism
L6	Rv3825c	4298772	4298816	178609	D277N		
L5	Rv3859c	4332530	4332574	178759	A1177G	Probable ferredoxin-dependent glutamate synthase	intermediary metabolism and respiration
L6	Rv3874	4352304	4352363	34928	Q28P	10 kDa culture filtrate antigen	cell wall and cell processes

						EsxB (LHP) (CFP10)	
L6	Rv3878	4357236	4357295	20015	G185A	ESX-1 secretion-associated protein EspJ	cell wall and cell processes
L5	Rv3879c	4357713	4357760	144864	A685T & A684D	ESX-1 secretion-associated protein EspK	cell wall and cell processes
L6	Rv3879c	4359339	4359377	120976	T143S		
L5	Rv3883c	4364689	4364757	144921	A12V	Membrane-anchored mycosin MycP1	intermediary metabolism and respiration
L6	Rv3883c	4363603	4363641	120428	L375P		

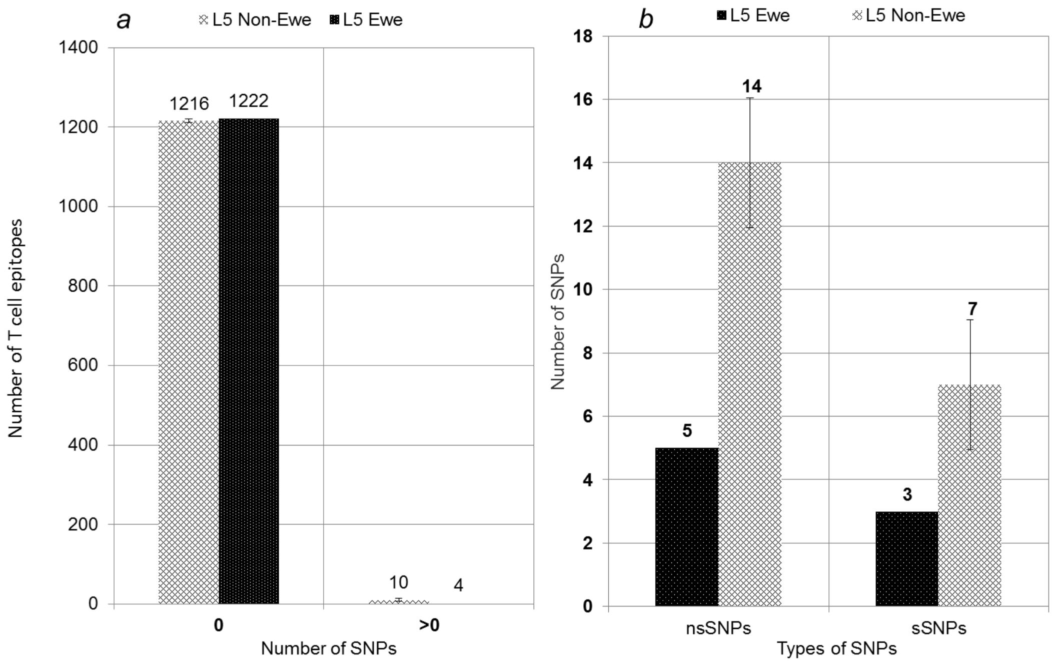

Supplementary figure S6: Number of epitopes with and without nsSNPs stratified by Lineage 5 with patient ethnicity (*a*). Number of non-redundant SNPs in epitopes stratified by ethnicity of L5 infected patients (*b*). (The values for the non-Ewe associated L5 are mean estimates of 10 random samples of size 15 with replacement)

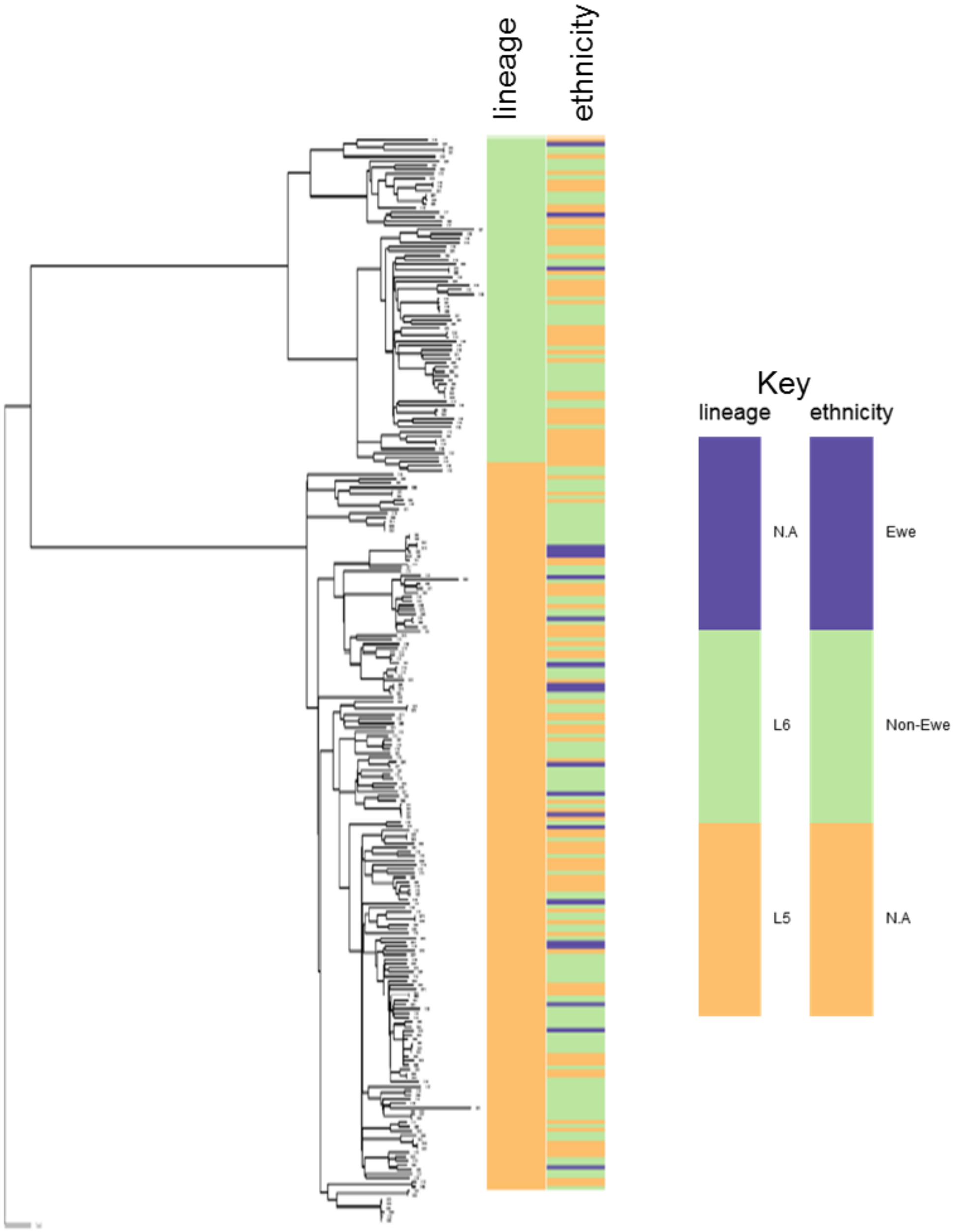

Supplementary figure S7: Uniform distribution of L5 isolated from ethnic Ewe TB patients among the L5 clade.

Supplementary data S8: Genomes and accession numbers of *Maf* from Ghana.

Genome Number	Isolate Name	Sequencing Name	Isolation Institute	Sequencing Institute	Provider	Accession Number	Country of Origin
G00568	N1194	N1194	NMIMR	STPH	SG	ERR841438	Ghana
G00572	N1201	N1201	NMIMR	STPH	SG	ERR841440	Ghana
G00573	N1202	N1202	NMIMR	STPH	SG	ERR841441	Ghana
G00731	N1203	N1203	NMIMR	STPH	SG	ERR2181457	Ghana
G00895	N1172	DY20	NMIMR	STPH	SG	ERR234202	Ghana
G00896	N1179	DY21	NMIMR	STPH	SG	ERR234113	Ghana
G00898	N1186	DY26	NMIMR	STPH	SG	ERR234204	Ghana
G00962	TBNM0176	N1200	NMIMR	ETH	SG	ERR2181458	Ghana
G03829	TBNM2359A	SNOG240	NMIMR	WTSI	DYM	ERR1334053	Ghana
G03830	TBNM3650	SNOG239	NMIMR	WTSI	DYM	ERR1334052	Ghana
G04127	TBNM2351	SNOG236	NMIMR	WTSI	DYM	ERR1334051	Ghana
G04128	TBNM2111	SNOG234	NMIMR	WTSI	DYM	ERR1334050	Ghana
G04129	TBNM3612	SNOG226	NMIMR	WTSI	DYM	ERR1334049	Ghana
G04130	TBNM2240B	SNOG224	NMIMR	WTSI	DYM	ERR1215483	Ghana
G04131	TBNM2036	SNOG223	NMIMR	WTSI	DYM	ERR1215482	Ghana
G04132	TBNM2096	SNOG222	NMIMR	WTSI	DYM	ERR1215481	Ghana
G04133	TBNM1510	SNOG221	NMIMR	WTSI	DYM	ERR1215480	Ghana
G04134	TBNM2273	SNOG218	NMIMR	WTSI	DYM	ERR1215479	Ghana
G04163	TBNM1821B	SNOG217	NMIMR	WTSI	DYM	ERR1215478	Ghana
G04164	TBNM1642	SNOG214	NMIMR	WTSI	DYM	ERR1215477	Ghana
G04165	TBNM2263	SNOG213	NMIMR	WTSI	DYM	ERR1215476	Ghana
G04166	TBNM1203	SNOG212	NMIMR	WTSI	DYM	ERR1215475	Ghana
G08466	TBNM1347	SNOG170	NMIMR	WTSI	DYM	ERR1082113	Ghana
G08467	TBNM1108	SNOG171	NMIMR	WTSI	DYM	ERR1082114	Ghana
G08468	TBNM2708	SNOG172	NMIMR	WTSI	DYM	ERR1082115	Ghana
G08469	TBNM2287	SNOG173	NMIMR	WTSI	DYM	ERR1082116	Ghana
G08470	TBNM2663	SNOG174	NMIMR	WTSI	DYM	ERR1082117	Ghana
G08471	TBNM2919	SNOG176	NMIMR	WTSI	DYM	ERR1082119	Ghana
G08472	TBNM2660	SNOG177	NMIMR	WTSI	DYM	ERR1082120	Ghana
G08473	TBNM3342	SNOG179	NMIMR	WTSI	DYM	ERR1082122	Ghana
G08474	TBNM3323	SNOG180	NMIMR	WTSI	DYM	ERR1082123	Ghana
G08475	TBNM2910	SNOG181	NMIMR	WTSI	DYM	ERR1082124	Ghana
G08476	TBNM1962	SNOG182	NMIMR	WTSI	DYM	ERR1082125	Ghana
G08477	TBNM3285	SNOG185	NMIMR	WTSI	DYM	ERR1082126	Ghana
G08478	TBNM1571	SNOG190	NMIMR	WTSI	DYM	ERR1082127	Ghana
G08479	TBNM1672	SNOG191	NMIMR	WTSI	DYM	ERR1082128	Ghana
G08480	TBNM1135	SNOG192	NMIMR	WTSI	DYM	ERR1082129	Ghana
G08481	TBNM1821	SNOG193	NMIMR	WTSI	DYM	ERR1082130	Ghana
G08482	TBNM2541	SNOG201	NMIMR	WTSI	DYM	ERR1082131	Ghana
G08483	TBNM1225	SNOG202	NMIMR	WTSI	DYM	ERR1082132	Ghana

G08484	TBNM1793	SNOG203	NMIMR	WTSI	DYM	ERR1082133	Ghana
G08485	TBNM2199	SNOG204	NMIMR	WTSI	DYM	ERR1082134	Ghana
G08486	TBNM2238B	SNOG205	NMIMR	WTSI	DYM	ERR1082135	Ghana
G08487	TBNM1582	SNOG207	NMIMR	WTSI	DYM	ERR1082136	Ghana
G08488	TBNM2026	SNOG208	NMIMR	WTSI	DYM	ERR1082137	Ghana
G08489	TBNM1529	SNOG209	NMIMR	WTSI	DYM	ERR1082138	Ghana
G08490	TBNM2384	SNOG211	NMIMR	WTSI	DYM	ERR1082139	Ghana
G08491	TBNM1573	SNOG215	NMIMR	WTSI	DYM	ERR1082140	Ghana
G08492	TBNM1280	SNOG216	NMIMR	WTSI	DYM	ERR1082141	Ghana
G08493	TBNM2464	SNOG219	NMIMR	WTSI	DYM	ERR1082142	Ghana
G08494	TBNM2604	SNOG220	NMIMR	WTSI	DYM	ERR1082143	Ghana
G08495	TBNM3359C	SNOG227	NMIMR	WTSI	DYM	ERR1203053	Ghana
G08496	TBNM3577	SNOG228	NMIMR	WTSI	DYM	ERR1203054	Ghana
G08497	TBNM2359B	SNOG229	NMIMR	WTSI	DYM	ERR1203055	Ghana
G08498	TBNM2319	SNOG210	NMIMR	WTSI	DYM	ERR1215474	Ghana
G08499	TBNM2541	SNOG230	NMIMR	WTSI	DYM	ERR1203056	Ghana
G08500	TBNM3030	SNOG231	NMIMR	WTSI	DYM	ERR1203057	Ghana
G08501	TBNM2423	SNOG232	NMIMR	WTSI	DYM	ERR1203058	Ghana
G08502	TBNM2266	SNOG233	NMIMR	WTSI	DYM	ERR1203059	Ghana
G08503	TBNM2048	SNOG237	NMIMR	WTSI	DYM	ERR1203061	Ghana
G08504	TBNM3530	SNOG238	NMIMR	WTSI	DYM	ERR1203062	Ghana
G08507	TBNM2834	SNOG243	NMIMR	WTSI	DYM	ERR1203065	Ghana
G08508	TBNM2667	SNOG244	NMIMR	WTSI	DYM	ERR1203066	Ghana
G08510	TBNM2710	SNOG246	NMIMR	WTSI	DYM	ERR1203068	Ghana
G08511	TBNM2608	SNOG247	NMIMR	WTSI	DYM	ERR1203069	Ghana
G08512	TBNM2519	SNOG248	NMIMR	WTSI	DYM	ERR1203070	Ghana
G08514	TBNM2878	SNOG250	NMIMR	WTSI	DYM	ERR1203072	Ghana
G08516	TBNM2890	SNOG252	NMIMR	WTSI	DYM	ERR1203074	Ghana
G08518	TBNM2698	SNOG254	NMIMR	WTSI	DYM	ERR1203076	Ghana
G08519	TBNM2874	SNOG255	NMIMR	WTSI	DYM	ERR1203077	Ghana
G08611	TBNM0039	SNOG003	NMIMR	WTSI	DYM	ERR502471	Ghana
G08612	TBNM0074	SNOG007	NMIMR	WTSI	DYM	ERR502475	Ghana
G08613	TBNM0076	SNOG008	NMIMR	WTSI	DYM	ERR502476	Ghana
G08614	TBNM0123	SNOG009	NMIMR	WTSI	DYM	ERR502477; ERR702399	Ghana
G08615	TBNM0443	SNOG014	NMIMR	WTSI	DYM	ERR502480	Ghana
G08616	TBNM0559	SNOG021	NMIMR	WTSI	DYM	ERR502487	Ghana
G08618	TBNM0596	SNOG025	NMIMR	WTSI	DYM	ERR502491; ERR702401	Ghana
G08619	TBNM0637	SNOG028	NMIMR	WTSI	DYM	ERR502494; ERR702402	Ghana
G08622	TBNM0712	SNOG034	NMIMR	WTSI	DYM	ERR502500	Ghana
G08623	TBNM0730	SNOG035	NMIMR	WTSI	DYM	ERR502501	Ghana
G08624	TBNM0736	SNOG036	NMIMR	WTSI	DYM	ERR502502	Ghana
G08625	TBNM0738	SNOG037	NMIMR	WTSI	DYM	ERR502503; ERR702403	Ghana
G08627	TBNM0750	SNOG039	NMIMR	WTSI	DYM	ERR502505	Ghana

G08628	TBNM0758	SNOG040	NMIMR	WTSI	DYM	ERR502506	Ghana
G08629	TBNM0767	SNOG041	NMIMR	WTSI	DYM	ERR502507; ERR702404	Ghana
G08630	TBNM0789	SNOG042	NMIMR	WTSI	DYM	ERR502508	Ghana
G08631	TBNM0791	SNOG043	NMIMR	WTSI	DYM	ERR502509	Ghana
G08632	TBNM1071	SNOG046	NMIMR	WTSI	DYM	ERR502512	Ghana
G08633	TBNM1079	SNOG047	NMIMR	WTSI	DYM	ERR502513	Ghana
G08634	TBNM1102	SNOG048	NMIMR	WTSI	DYM	ERR502514	Ghana
G08635	TBNM1115	SNOG049	NMIMR	WTSI	DYM	ERR502515	Ghana
G08636	TBNM1123	SNOG050	NMIMR	WTSI	DYM	ERR502516	Ghana
G08637	TBNM1144	SNOG051	NMIMR	WTSI	DYM	ERR502517	Ghana
G08641	TBNM1192	SNOG056	NMIMR	WTSI	DYM	ERR502521	Ghana
G08642	TBNM1200	SNOG057	NMIMR	WTSI	DYM	ERR502522	Ghana
G08643	TBNM1219	SNOG058	NMIMR	WTSI	DYM	ERR502523	Ghana
G08644	TBNM1257	SNOG060	NMIMR	WTSI	DYM	ERR502525	Ghana
G08646	TBNM1283a	SNOG062	NMIMR	WTSI	DYM	ERR502527	Ghana
G08647	TBNM1289	SNOG063	NMIMR	WTSI	DYM	ERR502528	Ghana
G08649	TBNM1316	SNOG065	NMIMR	WTSI	DYM	ERR502530	Ghana
G08650	TBNM1434	SNOG068	NMIMR	WTSI	DYM	ERR502533	Ghana
G08651	TBNM1697	SNOG069	NMIMR	WTSI	DYM	ERR502534	Ghana
G08652	TBNM1916	SNOG073	NMIMR	WTSI	DYM	ERR502536	Ghana
G08653	TBNM1918	SNOG074	NMIMR	WTSI	DYM	ERR502537	Ghana
G08655	TBNM0010	SNOG001	NMIMR	WTSI	DYM	ERR517471	Ghana
G08656	TBNM1950	SNOG206	NMIMR	WTSI	DYM	ERR1215473	Ghana
G08657	TBNM2988	SNOG200	NMIMR	WTSI	DYM	ERR1215472	Ghana
G08658	TBNM2151	SNOG199	NMIMR	WTSI	DYM	ERR1215471	Ghana
G08659	TBNM2319	SNOG198	NMIMR	WTSI	DYM	ERR1215470	Ghana
G08660	TBNM1343	SNOG076	NMIMR	WTSI	DYM	ERR702407	Ghana
G08661	TBNM1803	SNOG077	NMIMR	WTSI	DYM	ERR702408	Ghana
G08662	TBNM1818	SNOG078	NMIMR	WTSI	DYM	ERR702409	Ghana
G08663	TBNM1615	SNOG079	NMIMR	WTSI	DYM	ERR702410	Ghana
G08664	TBNM1291	SNOG080	NMIMR	WTSI	DYM	ERR702411	Ghana
G08665	TBNM1186	SNOG081	NMIMR	WTSI	DYM	ERR702412	Ghana
G08666	TBNM1309	SNOG082	NMIMR	WTSI	DYM	ERR702413	Ghana
G08667	TBNM1966	SNOG083	NMIMR	WTSI	DYM	ERR702414	Ghana
G08668	TBNM1733	SNOG084	NMIMR	WTSI	DYM	ERR702415	Ghana
G08669	TBNM1404	SNOG085	NMIMR	WTSI	DYM	ERR702416	Ghana
G08670	TBNM1726	SNOG086	NMIMR	WTSI	DYM	ERR702417	Ghana
G08671	TBNM1313	SNOG087	NMIMR	WTSI	DYM	ERR702418	Ghana
G08672	TBNM1452	SNOG088	NMIMR	WTSI	DYM	ERR702419	Ghana
G08673	TBNM1391	SNOG089	NMIMR	WTSI	DYM	ERR702420	Ghana
G08674	TBNM1158	SNOG090	NMIMR	WTSI	DYM	ERR702421	Ghana
G08675	TBNM1723	SNOG091	NMIMR	WTSI	DYM	ERR702422	Ghana
G08676	TBNM1741	SNOG092	NMIMR	WTSI	DYM	ERR702423	Ghana
G08677	TBNM1465	SNOG093	NMIMR	WTSI	DYM	ERR702424	Ghana
G08678	TBNM1082	SNOG094	NMIMR	WTSI	DYM	ERR702425	Ghana

G08679	TBNM1297A	SNOG095	NMIMR	WTSI	DYM	ERR702426	Ghana
G08680	TBNM1861A	SNOG096	NMIMR	WTSI	DYM	ERR702427	Ghana
G08681	TBNM0205	SNOG098	NMIMR	WTSI	DYM	ERR702428	Ghana
G08682	TBNM0006	SNOG100	NMIMR	WTSI	DYM	ERR702430	Ghana
G08683	TBNM1050	SNOG101	NMIMR	WTSI	DYM	ERR702431	Ghana
G08684	TBNM0132	SNOG102	NMIMR	WTSI	DYM	ERR702432	Ghana
G08686	TBNM1045	SNOG104	NMIMR	WTSI	DYM	ERR702434	Ghana
G08687	TBNM1841	SNOG105	NMIMR	WTSI	DYM	ERR702435	Ghana
G08688	TBNM1779	SNOG106	NMIMR	WTSI	DYM	ERR702436	Ghana
G08690	TBNM2080	SNOG109	NMIMR	WTSI	DYM	ERR751290	Ghana
G08691	TBNM1726B	SNOG110	NMIMR	WTSI	DYM	ERR751291	Ghana
G08692	TBNM2240	SNOG111	NMIMR	WTSI	DYM	ERR751292	Ghana
G08693	TBNM1879	SNOG112	NMIMR	WTSI	DYM	ERR751293	Ghana
G08694	TBNM2016	SNOG113	NMIMR	WTSI	DYM	ERR751294	Ghana
G08695	TBNM2090	SNOG114	NMIMR	WTSI	DYM	ERR751295	Ghana
G08696	TBNM1186B	SNOG115	NMIMR	WTSI	DYM	ERR751296	Ghana
G08697	TBNM1957	SNOG116	NMIMR	WTSI	DYM	ERR751297	Ghana
G08698	TBNM1313B	SNOG117	NMIMR	WTSI	DYM	ERR751298	Ghana
G08699	TBNM1723B	SNOG118	NMIMR	WTSI	DYM	ERR751299	Ghana
G08700	TBNM1421	SNOG119	NMIMR	WTSI	DYM	ERR751300	Ghana
G08701	TBNM1763	SNOG120	NMIMR	WTSI	DYM	ERR751301	Ghana
G08702	TBNM1391B	SNOG121	NMIMR	WTSI	DYM	ERR751302	Ghana
G08703	TBNM1779B	SNOG122	NMIMR	WTSI	DYM	ERR751303	Ghana
G08704	TBNM1814	SNOG123	NMIMR	WTSI	DYM	ERR751304	Ghana
G08705	TBNM1838	SNOG124	NMIMR	WTSI	DYM	ERR751305	Ghana
G08706	TBNM1875	SNOG125	NMIMR	WTSI	DYM	ERR751306	Ghana
G08707	TBNM1802	SNOG126	NMIMR	WTSI	DYM	ERR751307	Ghana
G08708	TBNM2010	SNOG127	NMIMR	WTSI	DYM	ERR751308	Ghana
G08709	TBNM2150	SNOG128	NMIMR	WTSI	DYM	ERR751309	Ghana
G08710	TBNM1818B	SNOG129	NMIMR	WTSI	DYM	ERR751310	Ghana
G08711	TBNM2196	SNOG130	NMIMR	WTSI	DYM	ERR751311	Ghana
G08712	TBNM1786	SNOG131	NMIMR	WTSI	DYM	ERR751312	Ghana
G08713	TBNM2163	SNOG132	NMIMR	WTSI	DYM	ERR751313	Ghana
G08714	TBNM1803B	SNOG133	NMIMR	WTSI	DYM	ERR751314	Ghana
G08715	TBNM2195	SNOG134	NMIMR	WTSI	DYM	ERR751315	Ghana
G08717	TBNM1044	SNOG138	NMIMR	WTSI	DYM	ERR751319	Ghana
G08718	TBNM1841B	SNOG139	NMIMR	WTSI	DYM	ERR751320	Ghana
G08719	TBNM1320	SNOG140	NMIMR	WTSI	DYM	ERR751321	Ghana
G08720	TBNM1984	SNOG141	NMIMR	WTSI	DYM	ERR751322	Ghana
G08721	TBNM1897	SNOG142	NMIMR	WTSI	DYM	ERR751323	Ghana
G08722	TBNM1926	SNOG143	NMIMR	WTSI	DYM	ERR751324	Ghana
G08723	TBNM1165	SNOG146	NMIMR	WTSI	DYM	ERR751327	Ghana
G08724	TBNM1383	SNOG147	NMIMR	WTSI	DYM	ERR751328	Ghana
G08725	TBNM0738B	SNOG148	NMIMR	WTSI	DYM	ERR751329	Ghana
G08726	TBNM0132B	SNOG149	NMIMR	WTSI	DYM	ERR751330	Ghana
G08727	TBNM1158B	SNOG150	NMIMR	WTSI	DYM	ERR751331	Ghana

G08728	TBNM0596C	SNOG151	NMIMR	WTSI	DYM	ERR751332	Ghana
G08729	TBNM1010	SNOG152	NMIMR	WTSI	DYM	ERR751333	Ghana
G08730	TBNM1048	SNOG154	NMIMR	WTSI	DYM	ERR751334	Ghana
G08731	TBNM2297	SNOG155	NMIMR	WTSI	DYM	ERR751335	Ghana
G08732	TBNM0123B	SNOG156	NMIMR	WTSI	DYM	ERR751336	Ghana
G08733	TBNM1166	SNOG157	NMIMR	WTSI	DYM	ERR751337	Ghana
G08734	TBNM0637B	SNOG158	NMIMR	WTSI	DYM	ERR751338	Ghana
G08735	TBNM1311	SNOG159	NMIMR	WTSI	DYM	ERR751339	Ghana
G08737	TBNM1082B	SNOG161	NMIMR	WTSI	DYM	ERR751341	Ghana
G08738	TBNM1045B	SNOG162	NMIMR	WTSI	DYM	ERR751342	Ghana
G08739	TBNM1012	SNOG163	NMIMR	WTSI	DYM	ERR751343	Ghana
G08740	TBNM1050B	SNOG164	NMIMR	WTSI	DYM	ERR751344	Ghana
G08741	TBNM1193	SNOG165	NMIMR	WTSI	DYM	ERR751345	Ghana
G08742	TBNM1030	SNOG166	NMIMR	WTSI	DYM	ERR751346	Ghana
G08743	TBNM2309	SNOG168	NMIMR	WTSI	DYM	ERR751348	Ghana
G08744	TBNM1291B	SNOG169	NMIMR	WTSI	DYM	ERR751349	Ghana
G08842	TBNM2035	SNOG197	NMIMR	WTSI	DYM	ERR1215469	Ghana
G08843	TBNM2556	SNOG196	NMIMR	WTSI	DYM	ERR1215468	Ghana
G08844	TBNM2238A	SNOG195	NMIMR	WTSI	DYM	ERR1215467	Ghana
G08845	TBNM2341	SNOG194	NMIMR	WTSI	DYM	ERR1215466	Ghana
G08846	TBNM2267	SNOG189	NMIMR	WTSI	DYM	ERR1215465	Ghana
G08847	TBNM2351	SNOG188	NMIMR	WTSI	DYM	ERR1215464	Ghana
G08848	TBNM1011	SNOG187	NMIMR	WTSI	DYM	ERR1215463	Ghana
G08849	TBNM1623	SNOG186	NMIMR	WTSI	DYM	ERR1215462	Ghana
G08850	TBNM1083	SNOG184	NMIMR	WTSI	DYM	ERR1215461	Ghana
G08851	TBNM1533	SNOG183	NMIMR	WTSI	DYM	ERR1215460	Ghana
G08852	TBNM3388	SNOG178	NMIMR	WTSI	DYM	ERR1082121	Ghana
G08853	TBNM2517	SNOG175	NMIMR	WTSI	DYM	ERR1082118	Ghana
G08854	TBNM1343B	SNOG167	NMIMR	WTSI	DYM	ERR751347	Ghana
G08855	TBNM1188	SNOG153	NMIMR	WTSI	DYM	ERR845916	Ghana
G08856	TBNM0791B	SNOG145	NMIMR	WTSI	DYM	ERR751326	Ghana
G08857	TBNM2007	SNOG144	NMIMR	WTSI	DYM	ERR751325	Ghana
G08858	TBNM1358	SNOG137	NMIMR	WTSI	DYM	ERR751318	Ghana
G08859	TBNM1382	SNOG136	NMIMR	WTSI	DYM	ERR751317	Ghana
G08860	TBNM431B	SNOG108	NMIMR	WTSI	DYM	ERR909754	Ghana
G08861	TBNM0297	SNOG099	NMIMR	WTSI	DYM	ERR702429	Ghana
G08862	TBNM1860A	SNOG097	NMIMR	WTSI	DYM	ERR909753	Ghana
G08863	TBNM1416	SNOG067	NMIMR	WTSI	DYM	ERR502532; ERR702406	Ghana
G08864	TBNM1325	SNOG066	NMIMR	WTSI	DYM	ERR502531	Ghana
G08865	TBNM1244	SNOG059	NMIMR	WTSI	DYM	ERR502524	Ghana
G08866	TBNM0802b	SNOG045	NMIMR	WTSI	DYM	ERR502511	Ghana
G08867	TBNM0802	SNOG044	NMIMR	WTSI	DYM	ERR502510; ERR702405	Ghana
G08868	TBNM0703	SNOG032	NMIMR	WTSI	DYM	ERR502498	Ghana
G08869	TBNM0701	SNOG031	NMIMR	WTSI	DYM	ERR502497	Ghana
G08870	TBNM0693	SNOG030	NMIMR	WTSI	DYM	ERR502496	Ghana

G08871	TBNM0629	SNOG027	NMIMR	WTSI	DYM	ERR502493	Ghana
G08872	TBNM0620	SNOG026	NMIMR	WTSI	DYM	ERR502492	Ghana
G08873	TBNM0588	SNOG024	NMIMR	WTSI	DYM	ERR502490	Ghana
G08874	TBNM0585b	SNOG023	NMIMR	WTSI	DYM	ERR502489	Ghana
G08875	TBNM0545b	SNOG020	NMIMR	WTSI	DYM	ERR502486	Ghana
G08876	TBNM0545	SNOG019	NMIMR	WTSI	DYM	ERR502485	Ghana
G08877	TBNM0530	SNOG018	NMIMR	WTSI	DYM	ERR502484	Ghana
G08878	TBNM0517	SNOG017	NMIMR	WTSI	DYM	ERR502483	Ghana
G08879	TBNM0459	SNOG016	NMIMR	WTSI	DYM	ERR502482	Ghana
G08880	TBNM0451b	SNOG015	NMIMR	WTSI	DYM	ERR502481	Ghana
G08881	TBNM0431	SNOG012	NMIMR	WTSI	DYM	ERR517472; ERR702400	Ghana
G08882	TBNM0408	SNOG011	NMIMR	WTSI	DYM	ERR502479	Ghana
G08883	TBNM0320	SNOG010	NMIMR	WTSI	DYM	ERR502478	Ghana
G08884	TBNM0070	SNOG006	NMIMR	WTSI	DYM	ERR502474	Ghana
G08885	TBNM0067	SNOG005	NMIMR	WTSI	DYM	ERR502473	Ghana
G08886	TBNM0064	SNOG004	NMIMR	WTSI	DYM	ERR502472	Ghana
G08887	TBNM0011	SNOG002	NMIMR	WTSI	DYM	ERR502470	Ghana
G11441	TBNM3293	SNOG225	NMIMR	WTSI	DYM	ERR1203052	Ghana
G11553	10448-01	lib3294	RCB	RCB	SN	Pending	Ghana
G11554	TBNM2859	SNOG235	NMIMR	WTSI	DYM	ERR1203060	Ghana
G11555	10449-01	lib3295	RCB	RCB	SN	ERR1203074	Ghana
G11556	10451-01	lib3296	RCB	RCB	SN	Pending	Ghana
G11557	2569-02	lib3329	RCB	RCB	SN	Pending	Ghana
G11558	2574-02	lib3330	RCB	RCB	SN	Pending	Ghana
G11559	2586-02	lib3331	RCB	RCB	SN	Pending	Ghana
G11560	2598-02	lib3332	RCB	RCB	SN	Pending	Ghana
G11561	2607-02	lib3333	RCB	RCB	SN	Pending	Ghana
G11565	3377-03	lib3388	RCB	RCB	SN	Pending	Ghana
G11566	3482-03	lib3334	RCB	RCB	SN	Pending	Ghana
G11577	4802-03	lib3338	RCB	RCB	SN	Pending	Ghana
G11578	4804-03	lib3339	RCB	RCB	SN	Pending	Ghana
G11580	5348-02	lib3340	RCB	RCB	SN	Pending	Ghana
G11582	5355-02	lib3341	RCB	RCB	SN	Pending	Ghana
G11583	5358-02	lib3342	RCB	RCB	SN	Pending	Ghana
G11584	5360-02	lib3343	RCB	RCB	SN	Pending	Ghana
G11585	5361-02	lib3344	RCB	RCB	SN	Pending	Ghana
G11586	5362-02	lib3345	RCB	RCB	SN	Pending	Ghana
G11587	5372-02	lib3346	RCB	RCB	SN	Pending	Ghana
G11588	5374-02	lib3347	RCB	RCB	SN	Pending	Ghana
G11589	5375-02	lib3348	RCB	RCB	SN	Pending	Ghana
G11590	5376-02	lib3349	RCB	RCB	SN	Pending	Ghana
G11591	5378-02	lib3350	RCB	RCB	SN	Pending	Ghana
G11592	5382-02	lib3351	RCB	RCB	SN	Pending	Ghana
G11593	5383-02	lib3352	RCB	RCB	SN	Pending	Ghana
G11594	5389-02	lib3353	RCB	RCB	SN	Pending	Ghana

G11595	5398-02	lib3354	RCB	RCB	SN	Pending	Ghana
G11596	5403-02	lib3355	RCB	RCB	SN	Pending	Ghana
G11597	5404-02	lib3356	RCB	RCB	SN	Pending	Ghana
G11598	5405-02	lib3357	RCB	RCB	SN	Pending	Ghana
G11599	5415-02	lib3358	RCB	RCB	SN	Pending	Ghana
G11600	5417-02	lib3359	RCB	RCB	SN	Pending	Ghana
G11601	5418-02	lib3360	RCB	RCB	SN	Pending	Ghana
G11602	5419-02	lib3361	RCB	RCB	SN	Pending	Ghana
G11603	5421-02	lib3362	RCB	RCB	SN	Pending	Ghana
G11604	5432-02	lib3363	RCB	RCB	SN	Pending	Ghana
G11605	5434-02	lib1185	RCB	RCB	SN	ERR552588	Ghana
G11606	5436-02	lib3364	RCB	RCB	SN	Pending	Ghana
G11607	5438-02	lib3365	RCB	RCB	SN	Pending	Ghana
G11608	5441-04	lib3389	RCB	RCB	SN	Pending	Ghana
G11609	5444-02	lib3366	RCB	RCB	SN	Pending	Ghana
G11610	5446-02	lib3367	RCB	RCB	SN	Pending	Ghana
G11611	5447-02	lib3368	RCB	RCB	SN	Pending	Ghana
G11612	5450-02	lib3369	RCB	RCB	SN	Pending	Ghana
G11613	5451-02	lib3370	RCB	RCB	SN	Pending	Ghana
G11614	5456-02	lib3371	RCB	RCB	SN	Pending	Ghana
G11615	5467-02	lib3372	RCB	RCB	SN	Pending	Ghana
G11616	5468-02	lib1191	RCB	RCB	SN	ERR552963	Ghana
G11618	5471-02	lib3374	RCB	RCB	SN	Pending	Ghana
G11619	5473-02	lib3375	RCB	RCB	SN	Pending	Ghana
G11620	5475-02	lib3376	RCB	RCB	SN	Pending	Ghana
G11621	5495-02	lib3377	RCB	RCB	SN	Pending	Ghana
G11623	6361-03	lib3283	RCB	RCB	SN	Pending	Ghana
G11626	8270-03	lib3285	RCB	RCB	SN	Pending	Ghana
G11627	8303-02	lib3380	RCB	RCB	SN	Pending	Ghana
G11637	9858-03	lib3286	RCB	RCB	SN	Pending	Ghana
G11640	10452-01	lib3297	RCB	RCB	SN	Pending	Ghana
G11641	10455-01	lib3298	RCB	RCB	SN	Pending	Ghana
G11642	10456-01	lib3299	RCB	RCB	SN	Pending	Ghana
G11643	10463-02	lib3384	RCB	RCB	SN	Pending	Ghana
G11644	10473-01	lib1186	RCB	RCB	SN	ERR552172	Ghana
G11645	10476-01	lib3300	RCB	RCB	SN	Pending	Ghana
G11646	10480-01	lib3301	RCB	RCB	SN	Pending	Ghana
G11647	10482-01	lib3385	RCB	RCB	SN	Pending	Ghana
G11648	10485-01	lib3302	RCB	RCB	SN	Pending	Ghana
G11649	10494-01	lib1187	RCB	RCB	SN	ERR552187	Ghana
G11650	10512-01	lib3304	RCB	RCB	SN	Pending	Ghana
G11651	10514-01	lib1189	RCB	RCB	SN	ERR552021	Ghana
G11652	10516-01	lib3305	RCB	RCB	SN	Pending	Ghana
G11653	10517-01	lib1190	RCB	RCB	SN	ERR552328	Ghana
G11654	10519-01	lib3306	RCB	RCB	SN	Pending	Ghana
G11661	12184-03	lib3386	RCB	RCB	SN	Pending	Ghana

G11662	12322-03	lib3387	RCB	RCB	SN	Pending	Ghana
G11665	1407-02	lib3311	RCB	RCB	SN	Pending	Ghana
G11666	1410-02	lib3312	RCB	RCB	SN	Pending	Ghana
G11667	1413-02	lib3313	RCB	RCB	SN	Pending	Ghana
G11668	1426-02	lib3314	RCB	RCB	SN	Pending	Ghana
G11669	1431-02	lib3315	RCB	RCB	SN	Pending	Ghana
G11670	1432-02	lib3316	RCB	RCB	SN	Pending	Ghana
G11671	1437-02	lib3317	RCB	RCB	SN	Pending	Ghana
G11672	1440-02	lib3318	RCB	RCB	SN	Pending	Ghana
G11673	1443-02	lib1188	RCB	RCB	SN	ERR551617	Ghana
G11674	1447-02	lib3319	RCB	RCB	SN	Pending	Ghana
G11675	1449-02	lib3035	RCB	RCB	SN	ERR868539	Ghana
G11676	1452-02	lib3320	RCB	RCB	SN	Pending	Ghana
G11677	1457-02	lib3321	RCB	RCB	SN	Pending	Ghana
G11678	1462-02	lib3322	RCB	RCB	SN	Pending	Ghana
G11679	1465-02	lib3323	RCB	RCB	SN	Pending	Ghana
G11680	1467-02	lib3324	RCB	RCB	SN	Pending	Ghana
G11681	1468-02	lib3325	RCB	RCB	SN	Pending	Ghana
G11682	1472-02	lib3326	RCB	RCB	SN	Pending	Ghana
G11683	1473-02	lib1184	RCB	RCB	SN	ERR552506	Ghana
G11684	1474-02	lib3327	RCB	RCB	SN	Pending	Ghana
G11693	2568-02	lib3328	RCB	RCB	SN	Pending	Ghana
G11705	8163-02	lib3378	RCB	RCB	SN	Pending	Ghana
G11706	8214-03	lib3287	RCB	RCB	SN	Pending	Ghana
G11707	8236-02	lib3379	RCB	RCB	SN	Pending	Ghana

KEY:

NMIMR: Noguchi Memorial Institute for Medical Research, University of Ghana, Accra, Ghana.

WTSI: Wellcome Trust Sanger Institute, Genome Campus, University of Cambridge, United Kingdom

STPH: Swiss Tropical and Public Health Institute, Basel, Switzerland.

RCB: Research Center Borstel, Borstel, Germany.

ETH: Genomics Facility, Department of Biosystems Science and Engineering, ETH Zurich, Basel, Switzerland.
